# Socioeconomic and family influences on dental treatment needs among Brazilian underprivileged schoolchildren participating in a dental health program

**DOI:** 10.1186/1472-6831-13-56

**Published:** 2013-10-19

**Authors:** Cristina Martins Lisboa, Janice Simpson de Paula, Glaucia Maria Bovi Ambrosano, Antonio Carlos Pereira, Marcelo de Castro Meneghim, Karine Laura Cortellazzi, Fabiana Lima Vazquez, Fábio Luiz Mialhe

**Affiliations:** 1 Department of Community Dentistry, Piracicaba Dental School, University of Campinas–UNICAMP, P.O. BOX 52, 13414-903 Piracicaba, SP, Brazil

**Keywords:** Socioenvironmental aspects, Oral health, Access health services

## Abstract

**Background:**

The objective of this study was to compare the socioeconomic and family characteristics of underprivileged schoolchildren with and without curative dental needs participating in a dental health program.

**Methods:**

A random sample of 1411 of 8-to-10 year-old Brazilian schoolchildren was examined and two sample groups were included in the cross-sectional study: 544 presented curative dental needs and the other 867 schoolchildren were without curative dental needs. The schoolchildren were examined for the presence of caries lesions using the DMFT index and their parents were asked to answer questions about socioenvironmental characteristics of their families. Logistic regression models were adjusted estimating the Odds Ratios (OR), their 95% confidence intervals (CI), and significance levels.

**Results:**

After adjusting for potential confounders, it was found that families earning more than one Brazilian minimum wage, having fewer than four residents in the house, families living in homes owned by them, and children living with both biological parents were protective factors for the presence of dental caries, and consequently, curative dental needs.

**Conclusions:**

Socioeconomic status and family structure influences the curative dental needs of children from underprivileged communities. In this sense, dental health programs should plan and implement strategic efforts to reduce inequities in oral health status and access to oral health services of vulnerable schoolchildren and their families.

## Background

Oral health is intrinsically linked to general health and quality of life. The impact of oral diseases on individuals is reflected in their days lost at school and work, difficulty with eating, reduced self-esteem, poor quality of life, among other consequences [1,2].

According to a national survey conducted in Brazil in 2010, 18.1% of children aged 12 years had never been to the dentist, and of these, 60.8% reported curative dental treatment needs [3]. This situation indicates that there are inequalities in oral health and difficulties in access to dental care experienced by this population.

The benefits of access to dental treatment have been discussed in several articles, such as the study of Alkarimi et al. [4], who reported that the treatment of caries in schoolchildren improved their oral health conditions and satisfaction with their teeth, smile and appetite, which in turn, have a strong influence on their overall health. In addition, other studies have shown the impact of orthodontic treatment, periodontal treatment and early childhood caries treatment on the subjective perceptions of quality of life between schoolchildren and their parents [5-11].

Dental schools play an important role in promoting access to dental services for schoolchildren [12-18]. In Brazil, the Piracicaba Dental School has developed a project entitled “Program Always Smiling” (PAS) in partnership with public and private institutions, with the main objective of offering dental care to approximately 3,000 underprivileged children between 6 to 10 years of age from public schools in Piracicaba, São Paulo, Brazil each year. The dental care model proposed in the project comprises preventive and curative interventions with the aim of promoting the oral health of children and their families [19].

Although the influence of social determinants on oral health has been recognized in the literature [20,21], little is known about the differences in socioeconomic and family characteristics between schoolchildren with and without curative dental needs, belonging to poverty-income groups. Thus, the objective of this study was to investigate the socioeconomic and family characteristics of schoolchildren with and without curative dental needs, from poor families participating in a dental health program.

## Methods

### Ethical aspects

Before this study was conducted, the project was approved by the Research Ethics Committee (No. 111/2010) of the Piracicaba Dental School, University of Campinas. The inclusion of children and parents to participate in this study depended on obtaining written permission from the children’s parents/guardians, for this purpose.

### Sample

The city of Piracicaba has 55 primary schools with a total of 10,155 schoolchildren in the age group 8-10 years, enrolled in 2011. From the social exclusion index (SEI), an index elaborated by local government on the basis of social indicators of the city neighborhoods, it was possible to identify the schools belonging to the areas with high vulnerability, i.e., those with the worst SEI [22]. Of these, 9 public schools were randomly selected by the cluster sampling method. To calculate the sample size of the study, a power of 90% was considered, with an odds ratio of 1.5 and percentage response from the unexposed group of 35%, resulting in the selection 1411 children aged 8-10 years from Piracicaba, São Paulo, Brazil. Among them, 544 presented curative dental needs and were treated in the PAS and the other 867 schoolchildren were without curative dental needs.

The exclusion criteria were schoolchildren who were outside the stipulated age of 8 to 10 years; those with debilitated health; whose parents did not grant permission for participation in the study; or who did not responded satisfactorily to the questionnaire.

### Examination methodology

The data concerning the clinical characteristics of the schoolchildren attended by the PAS were obtained by dentists working in the municipal health system in partnership with the program. The dentists examined all children using a dental probe and mirror, under natural light in outdoor setting. Dental caries were registered using the DMFt and dmft indexes according to the World Health Organization recommendations [23]. Before data collection, practical and theoretical activities were performed in calibration exercises. Intra and inter-examiner reliability was assessed by Kappa statistics and a percentage agreement (higher than 0.85) was considered good.

### Questionnaire

Information about the socioenvironmental characteristics of the schoolchildren’s families were collected by means of a questionnaire sent to their parents. This instrument addressed issues related to socioeconomic characteristics (family income, parents’ educational level, home ownership, government assistance, parents’ occupation), and family environment (number of residents in the house, children living with both biological parents, schoolchildren’s caregivers outside of school hours). Data on the children’s gender were also collected. The questionnaire used was adapted from Paula et al. [2].

### Data analysis

Bivariate analyses using the Chi-square test (χ2) were performed to test the influence of independent variables on dependent variables. The independent variables were: monthly family income, based on the number of minimum wages which the family receives (≤ 1 and > 1 minimum wages), considering the Brazilian minimum wage (BMW) at time of data collection of approximately US$ 290 per month; parents’ educational level (up to 8 and more years of schooling); number of residents in the children’s house (up to 4 or more); home ownership (yes/no); family government assistance (yes/no), children living with both biological parents (yes/no), father’s occupation (unemployed/employee; mother’s occupation (housewife, employee); schoolchildren’s caregivers outside of school hours (parents/others), data that were dichotomized according other studies [24-26] and/or by the median. The dependent variable *treatment need* was dichotomized into '*with curative dental needs*’ and '*without curative dental needs’*. After this multiple logistic regression analyses using the stepwise procedure were performed in order to identify the risk indicators for treatment need. Only the independent variables with p value less than 0.20 were tested in the regression analysis in order to eliminate those that would make little contribution to the model, and those with p ≤ 0.05 remained in model after the adjustments. The logistic regression models were adjusted estimating the Odds Ratios (OR), their 95% confidence intervals (CI), and significance levels. All statistical tests were performed using the SAS software program *(SAS institute Inc 2001, version 9.2, Cary, North-Carolina/USA)*[27] at 5% significance level.

## Results

The sample was composed of a similar number of male (49%) and female (51%) subjects. As regards socioeconomic variables, 66.9% of families had an income ≤ 1 Brazilian minimum wages and most fathers (62.3%) and mothers (60.5%) had attended school for fewer than 8 years. With respect to family environment, 63.7% of children lived with both biological parents and 44.7% stayed with caregivers other than their parents outside school hours (Table 1).

**Table 1:** Characteristics of schoolchildren participating in the Program “Always Smiling”

**Variable**	**n**	**%**
Gender		
Male	701	49.0
Female	710	51.0
Monthly family income		
≤ 1 minimum wage*	385	66.9
> 1 minimum wage	942	33.1
Father’s education		
≤ 8 years	639	62.3
> 8 years	387	37.7
Mother’s education		
≤ 8 years	818	60.5
> 8 years	533	39.5
Number of residents in the house		
> 4 persons	698	50.7
≤ 4 persons	679	49.3
Home ownership		
No	556	40.2
Yes	827	59.8
Government assistance		
Yes	393	28.2
No	1000	71.8
Children living with both biological parents		
No	511	36.3
Yes	859	63.7
Father’s ocupation		
Unemployed	139	13.3
Employed	907	86.7
Mother’s ocupation		
Housewife	587	45.0
Employee	718	55.0
Schoolchildren’s caregivers outside of school hours		
Others	611	44.7
Father and/or Mother	755	55.3

Piracicaba, Brazil, 2011.

*Minimum wage at the time of data collection, approximately US$ 290.00.

With reference to caries prevalence in the treated schoolchildren, the dmft was 2.01 (SD = 2.06), DMFT was 0.44 (SD = 1.22), and 132 (24.2%) of the schoolchildren had more than one affected tooth (> 1 carious teeth).

Table 2 shows the association of independent variables with treatment need according to the Chi-square test. High monthly family income, father’s and mother’s high educational level, lower number of residents in the house, family living in home ownership properties, family without government assistance, children living with both biological parents and father and/or mother being the children’s caregivers outside school hours was associated with reduced dental treatment need (p ≤ 0.05).

**Table 2:** Bivariate analysis for association between treatment need and socioenvironmental profile

			**With curative dental need**		**Without curative dental need**				
**Variable**	**Categories**	**Total**	**N**	**%**	**n**	**%**	**OR**	**CI 95%**	**p**
Gender	Male	700	270	38.6	430	61.4	Ref		
	Female	711	274	38.5	437	61.5	1.00	0.8059-1.2373	0.9895
Monthly family income	≤ 1 minimum wage*	385	179	46.5	206	53.3	Ref		
	> 1 minimum wage	942	334	35.5	608	64.5	0.63	0.4970-0.8042	0.0002
Father’s education	≤ 8 years	639	255	40.0	384	60.0	Ref		
	> 8 years	387	124	32.0	263	68.0	0.71	0.5443-0.9262	0.0116
Mother’s education	≤ 8 years	818	337	41.0	481	59.0	Ref		
	> 8 years	533	186	35.0	347	65.0	0.76	0.610-0.959	0.0203
Number of residents in the house	> 4 persons	698	287	41.0	411	59.0	Ref		
	≤ 4 persons	679	244	36.0	435	64.0	0.80	0.646-0.998	0.0484
Home ownership	No	556	236	42.5	320	57.5	Ref		
	Yes	827	298	36.0	529	64.0	0.76	0.613-0.952	0.0164
Government assistance	Yes	393	180	45.9	213	54.1	Ref		
	No	1000	358	35.8	642	64.2	0.66	0.521-0.836	0.0006
Children living with both biological parents	No	511	225	44.0	286	56.0	Ref		
	Yes	859	302	35.1	557	64.9	0.69	0.551-0.862	0.0011
Father’s ocupation	Unemployed	139	61	43.8	78	56.2	Ref		
	Employed	907	323	35.6	584	64.4	0.71	0.493-1.015	0.0604
Mother’s ocupation	Housewife	587	208	35.5	379	64.5	Ref		
	employee	718	290	40.4	428	59.6	1.23	0.985-1.547	0.0669
Schoolchildren’s caregivers outside of school hours	Others	611	258	42.2	353	57.8	Ref		
	Father and/or Mother	755	264	35.0	491	65.0	0.74	0.591-0.916	0.0061

Piracicaba, Brazil, 2011.

*Minimum wage at the time of data collection, approximately US$ 290,00.

OR = Odds Ratio.

CI = Confidence Intervals.

Reference levels of dependent variable: treatment need.

All the variables with p < 0.20 were selected for the multiple logistic regression analysis. Among them, families earning more than one Brazilian minimum wage, having fewer than four residents in the house, families living in home-ownership properties and children living with both biological parents were protective factors for the presence of dental caries, and consequently, curative dental needs (Table 3). Therefore, children from families earning more than one minimum wage, residing with fewer people in the house, who live in their own home and with their biological parents showed less chance of having curative dental needs.

**Table 3:** Multiple logistic regression for association between treatment need and socioenvironmental profile

			**With curative dental need**		**Without curative dental need**				
		**Total**	**n**	**%**	**n**	**%**	**OR**	**CI 95%**	**p**
Monthly family income	≤ 1 minimum wage*	385	179	46.5	206	53.3	Ref		
	> 1 minimum wage	942	334	35.5	608	64.5	0.73	0.567-0.944	0.0162
Number of residents in the house	> 4 persons	698	287	41.0	411	59.0	Ref		
	≤ 4 persons	679	244	36.0	435	64.0	0.77	0.615-0.973	0.0281
Home ownership	No	556	236	42.5	320	57.5	Ref		
	Yes	827	298	36.0	529	64.0	0.78	0.623-0.994	0.0440
Children living with both biological parents	No	511	225	44.0	286	56.0	Ref		
	Yes	859	302	35.1	557	64.9	0.72	0.570-0.923	0.0091

Piracicaba, Brazil, 2011.

*Minimum wage at the time of data collection, approximately US$ 290,00.

OR = Odds Ratio.

CI = Confidence Intervals.

Reference levels of dependent variable: treatment need.

## Discussion

The results presented in this study revealed that there are social inequalities in the oral health of schoolchildren, even within a population with low socioeconomic status. Several researchers have emphasized the family environment and its socioeconomic conditions as mediators of health and disease in schoolchildren [28-32]. In the final logistic regression model, it was observed that children living in homes with a monthly family income of more than one Brazilian minimum wage, had less chance of presenting curative dental needs than their counterparts, a finding similar to that shown in the study of Paredes et al. [33]. Thus, even in underprivileged families, we found a deprivation gradient for dental caries experience and curative dental needs. It is known that underprivileged families have less access to broader and better health information, fewer resources to buy and replace oral hygiene aids, and fewer favorable conditions to make healthier choices, including dietary choices and access to dental care [29-34]. In addition, individuals in poor socioeconomic situations suffer from psychological and social problems because of living in poverty, influencing the way parents care for their children [35,36].

Children participating in PAS, who were living with fewer than 4 residents in the home, had less chance of having curative dental needs than those who lived with more than 4 residents in the home. Studies have shown that household overcrowding had an inverse relationship with healthy habits of nutrition and hygiene, oral health-related quality of life, and were predictors of traumatic dental injuries in children and adolescents [2,37-39]. Thus, overcrowding may have both direct and indirect effects on the general and oral health of the members of families, and poorer schoolchildren living in homes with fewer individuals was a protective factor for curative dental needs.

Home ownership, an environmental living condition, was another protective factor associated with fewer curative dental needs in schoolchildren, differing from the findings of Pereira et al. [40], which did not observe any associations with the DMFT index in 12 year-olds in the same city as the one of this study. However, their study sample was composed of children from public and private schools in Piracicaba, São Paulo, Brazil and the majority of their families had a monthly family income of over 2 Brazilian minimum wages. Studies have shown that home ownership may improve the psychological well-being of homeowners and support better parenting practices, which may lead to better child outcomes even in disadvantaged families [41-43]. Therefore, it is important that this variable be taken into account by health managers when planning their actions, in order to reduce inequities in oral health of this population, and increase its access to oral health services.

Family structures are changing globally and in Brazil, with an increasing number of non-nuclear and non-biological parents [44], studies have shown that family structure can have an impact on the oral health status, oral health-related quality of life, and self-perceived oral health of children and adolescents [2,29,45,46]. It was observed that underprivileged children living with both biological parents was a protective factor, as they presented fewer restorative dental treatment needs than those from non-nuclear families. The literature provides evidences that nuclear families were more likely to have a supportive economic and psychological environment for performing better health behaviors than the environment provided by single or separated parents. The latter are generally more stressed to earn enough income to sustain their children, resulting in negligent attitudes towards monitoring oral health and using dental services for both themselves and their children [29,34,45-47].

In addition to the direct impact of social determinants of health on children’s oral health, behavioral, psychological and social factors could also generate inequities of access to dental services, as poorer children were less likely to use these services [31,48-51]. As observed in the present study, most of the schoolchildren needing dental curative care were those living with families in worse economic and home environments, which highlight the importance of community dental health programs such as PAS to create mechanisms to improve access to and the use of dental services by those who most need them, thereby creating equity in access to health and not an “inverse care law” demand [48,52,53].

The literature presents several suggestions to increase dental attendance for children. Tellen et al. [50] point out that to encourage access to dental care for schoolchildren, it is necessary for mothers to incorporate the value of preventive and curative dental care into their children’s upbringing, especially in vulnerable populations. However, socioeconomic and psychological aspects of parents such as scheduling caregivers, transportation difficulties, fear of the dentist, provider availability, past satisfaction with dental care received, oral health beliefs, among other factors, could be a barrier that restrains/prevents the capacity of motivation from being transformed into action, impeding the access of low-income caregivers to oral health services for their children, and leading to them having a higher level of accumulated treatment needs [48,50,51].

The organization of the PAS project, unlike many dental programs, is based on the formation of a strategic network of institutional, financial and personnel support of key partners that enables continuous and comprehensive care for school children. The PAS project used its own transport donated by the Municipal Department of Education, to take schoolchildren to school and take on-site dental service to them, thereby facilitating access to dental care. All children are accompanied by educational monitors from participating schools. The goal of this strategy is to overcome the barriers imposed by the geographical location of services and indirect costs involved in transporting children to their dental treatment [54-56]. Moreover, the organization of PAS services during school hours allows greater accessibility to children’s dental care, since there is no need for parents to lose working hours to take their children for dental treatment [12]. In addition, this system facilitates the decision making of parents to take care of oral health needs of their children, instead of relying on the parents’ individual motivation to seek care for them. It is known that even in reimbursement systems there is no significant increase in their use by these groups [57].

Another important aspect of the PAS project is the participation of school teachers and principals in raising the awareness of parents about the importance of children’s participation in the program. According to Telleen et al. [50] mothers who had satisfactory communication with the dentist, who believed that dentist’s visits were for the purpose of keeping the child’s teeth healthy and believed in the importance of taking the child to the dentist regularly were more likely to return to the dental office regularly. Furthermore, dental screening at schools, performed by dentists participating in the program, helps with the detection of normative dental treatment needs that are often not detected by the guardians [14]. Similar programs have been developed in the UK and India demonstrating that the active search for cases of diseases in schools encourages access to dental care and awareness of both parents and children of the need for this, especially among low-income groups [58,59]. Therefore, health programs such as PAS, based on healthy alliances, and targeting resources to areas of greatest social exclusion are an essential requirement for tackling the oral health inequalities of children [60].

Despite the fact that dental services generally do not deal with the social determinants of health that affect oral diseases, it is known they have important impact on health inequalities when they improve accessibility and respond appropriately to the healthcare needs of different social groups [20]. Evidence has shown that the availability of a regular source of dental care was a strong predictor of dental visits in the past 12 months, among persons in a vulnerable population. Thus, improving access to oral health services could allow standardization of the risk profile of children from different sociodemographic backgrounds and impact significantly on the percentage of children requiring urgent dental treatment, and on the number of decayed teeth in children at low-income schools [49,54,61]. Therefore, it would be better for dental professionals to know the impact of socioenviromental conditions and family structure on the oral health of individuals, in order to plan intersectorial actions, as in the case of PAS, which positively impact the health of populations in a sustainable manner, especially those who are most vulnerable [62-65].

In spite of the significant results observed and discussed in this study, some limitations should be considered. It is a cross-sectional study, in which the causal relationship cannot be adequately assessed. Therefore, longitudinal follow-up is required for further insights into the impact of PAS on the reduction of inequalities in oral health of underprivileged schoolchildren.

## Conclusion

In conclusion, it was observed that socioeconomic status and family structure influences the curative dental needs of children from underprivileged communities. In this sense, dental care services based on the principles of the PAS project should be relevant to reduce inequities in oral health status and access to oral health services, positively impacting on the quality of life of vulnerable schoolchildren.

## Competing interests

The authors declare that they have no competing interests.

## Authors’ contributions

CML and JSP participated in the conception and design of the study, data interpretation, data acquisition, and drafting the manuscript. GMBA and KLC participated in data analyses. ACP and MCM contributed to critical revision of manuscript. FLV contributed to the data collection. FLM participated in the conception and design of the study and critical revision of manuscript. All authors read and approved the final manuscript.

## Pre-publication history

The pre-publication history for this paper can be accessed here:

http://www.biomedcentral.com/1472-6831/13/56/prepub
